# Supplementary material for: Differing Genetics of Saline and Cocaine Self‐Administration in the Hybrid Mouse Diversity Panel
Source: Genes Brain Behav. 2025 Jun 17;24(3):e70029. doi: 10.1111/gbb.70029 (PMC12173464; doi:10.1111/gbb.70029)
Supplement: Supplementary file 2 — Table S2. Significant loci for saline IVSA. [file GBB-24-e70029-s001.zip › Table S2 caption.docx]

“Table S2. Significant loci for saline IVSA.”
